# Study on the Mechanism of Liuwei Dihuang Pills in Treating Parkinson's Disease Based on Network Pharmacology

**DOI:** 10.1155/2021/4490081

**Published:** 2021-10-28

**Authors:** Dongtao Lin, Yudan Zeng, Deyu Tang, Yongming Cai

**Affiliations:** ^1^College of Public Health, Guangdong Pharmaceutical University, China; ^2^College of Medical Information Engineering, Guangdong Pharmaceutical University, China; ^3^Guangdong Provincial TCM Precision Medicine Big Data Engineering Technology Research Center, China

## Abstract

**Background:**

Parkinson's disease (PD) is a common neurodegenerative disease in middle-aged and elderly people. Liuwei Dihuang (LWDH) pills have a good effect on PD, but its mechanism remains unclear. Network pharmacology is the result of integrating basic theories and research methods of medicine, biology, computer science, bioinformatics, and other disciplines, which can systematically and comprehensively reflect the mechanism of drug intervention in disease networks.

**Methods:**

The main components and targets of herbs in LWDH pills were obtained through Traditional Chinese Medicine Systems Pharmacology Database and Analysis Platform (TCMSP). Its active components were screened based on absorption, distribution, metabolism, and excretion (ADME); the PD-related targets were obtained from the Genecards, OMIM, TTD, and DRUGBANK databases. We used R to take the intersection of LWDH- and PD-related targets and Cytoscape software to construct the drug-component-target network. Moreover, STRING and Cytoscape software was used to analyze protein–protein interactions (PPI), construct a PPI network, and explore potential protein functional modules in the network. The Metascape platform was used to perform KEGG pathway and GO function enrichment analyses. Finally, molecular docking was performed to verify whether the compound and target have good binding activity.

**Results:**

After screening and deduplication, 210 effective active ingredients, 204 drug targets, 4333 disease targets, and 162 drug-disease targets were obtained. We consequently constructed a drug-component-targets network and a PPI-drug-disease-targets network. The results showed that the hub components of LWDH pills were quercetin, stigmasterol, kaempferol, and beta-sitosterol; the hub targets were AKT1, VEGFA, and IL6. GO and KEGG enrichment analyses showed that these targets are involved in neuronal death, G protein-coupled amine receptor activity, reactive oxygen species metabolic processes, membrane rafts, MAPK signaling pathways, cellular senescence, and other biological processes. Molecular docking showed that the hub components were in good agreement with the hub targets.

**Conclusion:**

LWDH pills have implications for the treatment of PD since they contain several active components, target multiple ligands, and activate various pathways. The hub components possibly include quercetin, stigmasterol, kaempferol, and beta-sitosterol and act through pairing with hub targets, such as AKT1, VEGFA, and IL6, to regulate neuronal death, G protein-coupled amine receptor activity, reactive oxygen species metabolic process, membrane raft, MAPK signaling pathway, and cellular senescence for the treatment of PD.

## 1. Introduction

Parkinson's disease (PD) is a common neurological disorder in middle- and old-aged people. It is characterized by the progressive degeneration of dopaminergic neurons in the substantia nigra as well as pathological changes mediated by the formation of Lewy bodies, biochemical alterations by the decrease of dopamine (DA) transmitters in the striatum, and imbalance of DA and acetylcholine transmitters. PD is manifested as a multisymptomatic disorder, including symptoms such as tremor, myotonia, motor retardation, postural balance and sleep alterations, olfactory changes, autonomic nervous dysfunction, and cognitive and mental degeneration [1]. Epidemiological research showed that the prevalence of PD in people older than 60 years in Europe and the United States reached 1%, and over 4% were older than 80 years. The prevalence rate of PD in people over 65 years old in China is 1.7%, which is similar to that in European and American countries [2, 3]. Drug therapy is the first choice of management for PD, in which levodopa is the standard treatment and the most effective symptomatic drug [4]. However, levodopa cannot completely cure PD; the long-term efficacy of the drug gradually worsens and causes a series of side effects [5].

Traditional Chinese medicine (TCM) has been used for over 2,000 years for treating PD. TCM believes that kidney yin deficiency is a major key factor. Liuwei Dihuang (LWDH) pills are a classic kidney-tonifying prescription created by Qian Yi, a famous doctor during the Northern Song Dynasty. LWDH pills contain six herbal formulations: Rehmanniae radix Praeparata (Shudihuang, SDH), Rhizoma dioscoreae (Shanyao, SY), Cornus officinalis Sieb (Shanzhuyu, SZY), Poria cocos (Fuling, FL), Cortex moutan (Mudanpi, MDP), and Alisma orientale (Zexie, ZX). Clinical practice has proven that LWDH pills are effective in treating PD and can alleviate its autonomic symptoms. Zhong et al. [6] managed 53 patients in the treatment group with LWDH pills on the basis of conventional PD drug treatment and 36 patients in the control group with conventional PD drugs for 6 months. The results showed that the total effective rates of the treatment and control groups were 81% and 61.5%, respectively. The treatment group showed significantly better results than the control group treated with conventional Western medicine (*P* < 0.05). Fang et al. [7] randomly divided 128 patients with DA replacement therapy into two groups. The control group comprised 63 patients who received DA replacement therapy alone; the experimental group comprised 65 patients who received DA replacement therapy in combination with LWDH pills. The patients were evaluated on the SCOPA-AUT scale after 6 months of treatment. The results confirmed that LWDH pills can improve the autonomic symptoms of PD in many aspects, such as the urinary system, body temperature regulation, and sexual function. However, the six herbs in LWDH pills have complex chemical components, and their targets and mechanisms remain unclear.

Network pharmacology (NP) was first proposed by the British scholar, Andrew L. Hopkins, in 2007. NP is based on the rapid development of systems biology and multidirectional pharmacology. This was a new idea of drug design, which aims to expand available drugs [8]. It is based on the “disease-gene-target-drug” interaction network foundation, through the analysis of genes, proteins, diseases, drugs, and other information in existing databases, combined with existing research and the use of professional networks. Scientific and computational chemistry reveal the intervention and influence of drugs on the disease network, thereby showcasing the synergistic effect of drugs on the human body [9]. The holistic and systematic characteristics of its research strategy have the same goal as the theory of TCM in diagnosing and treating diseases from a holistic perspective. NP is suitable for studying the relationship between various drug components and disease targets in TCM and is, therefore, being widely used in TCM research. Li et al. [10] used HERB BIOMAP data collection, target map clustering, network target analysis, and other methods to determine the anti-diabetic activity of the TCM, Gegen Qinlian decoction. 4-Hydroxymethylphenytoin increased insulin secretion in RIN-5F cells and promoted 3T3-L1 fat cells. Zhang et al. [11] through molecular docking and network analysis clarified the main active compounds, targets of action, and various pharmacological mechanisms of Reduning injection in the treatment of upper respiratory tract infections. They reported that Reduning inhibited virus replication by directly acting on the key to regulating the life cycle of respiratory virus proteins and indirectly regulating the host immune system. Zeng et al. [12] conducted network pharmacological analysis on Chaihu Shugan Powder and screened out 152 active ingredients, including sapogenin F, sapogenin G, sapogenin C, leucoflorin, and hesperidin. Through GO and KEGG enrichment analyses, it was found that Chaihu Shugan Powder reduces Abeta-induced neuronal cell death and PC12 cell apoptosis through the PI3K-AKT signaling pathway, suggesting that it may have a therapeutic effect on Alzheimer's disease.

## 2. Materials and Methods

### 2.1. Collection of Compounds and Target Prediction of LWDH Pills

Traditional Chinese Medicine Systems Pharmacology Database and Analysis Platform (TCMSP, https://tcmspw.com/tcmsp.php) is an open-source database that specializes in analyzing TCM, integrating pharmacodynamics, pharmacokinetics, target prediction, and genomics [13]. We obtained the compounds of each herb in LWDH pills from TCMSP with the search terms, “Shudihuang,” “Shanyao,” “Shanzhuyu,” “Fuling,” “Mudanpi,” and “Zexie.” Before target prediction, absorption, distribution, metabolism, and excretion (ADME) were used to select biologically active compounds that contribute to its therapeutic effect, and those with poor pharmacological properties and poor drug capabilities are removed [14]. We chose oral bioavailability (OB) ≥ 30% and drug-likeness (DL) ≥ 0.18, as the ADME parameters. The target LWDH pills were also obtained through TCMSP; the selected active compounds were collected in respective target databases to relate to their target.

### 2.2. Predicting Target of PD

We obtained PD-related targets from four databases: (1) Drug Bank (https://go.drugbank.com/, version 5.1.8) [15], a comprehensive, free-to-access, online database containing information on drugs and drug targets; (2) Therapeutic Target Database (TTD, http://db.idrblab.net/ttd/, updated June 1, 2020) [16], which provides information about the known and expected therapeutic protein and nucleic acid targets, targeted disease, pathway information, and the corresponding drugs directed at each of these targets; (3) GeneCards (https://www.genecards.org/, version 5.0) [17], which provides detailed information about all genes that have been annotated and predicted by humans. It automatically integrates gene-centric data from approximately 100 data sources. This includes genome, transcriptome, proteome, genetics, and clinical and functional information; and (4) Online Mendelian Inheritance in Man (OMIM, https://omim.org/, updated January 19, 2021) [18], a comprehensive and authoritative compendium of human genes and genetic phenotypes that are freely available and updated daily. The full-text, referenced overviews in OMIM contain information on all known Mendelian disorders and over 15,000 genes. We used “Parkinson's disease” as a keyword to screen disease targets in each database and summarized the obtained targets and removed duplicate values; finally, UniProt (https://www.uniprot.org/) was used to standardize the obtained targets.

### 2.3. Construction and Analysis of Drug-Component-Target Network

In order to clarify the interaction between the targets of LWDH pills and the PD-related targets, R was used to select their intersection and draw the Venn diagram to obtain drug-disease targets, which were the targets of LWDH pills in the treatment of PD (disease targets). We screened out the active components that can target the drug targets and describe the relationship between LWDH pills, active components, and drug-disease targets and then imported into Cytoscape (https://cytoscape.org/, Version 3.8.0) to construct a drug-component-target network. Cytoscape is an open-source software platform for visualizing molecular interaction networks and biological pathways and integrating these networks with annotations, gene expression profiles, and other state data [19]. This software was used to analyze network parameters, including degree, betweenness centrality (BC), and closeness centrality (CC), to screen the hub components and targets of LWDH pills and the relationship between them.

### 2.4. Protein–Protein Interaction (PPI) Network Construction and Module Screening

STRING (https://string-db.org/, version 11.0) is a database of known and predicted PPIs [20]. The interactions include direct (physical) and indirect (functional) associations; they stem from computational prediction, knowledge transfer between organisms, and interactions aggregated from other (primary) databases. We submitted the targets in the intersection to STRING to identify information on PPIs and used Cytoscape to visualize the network. Moreover, to accurately analyze the action of LWDH pills in treating PD, it is necessary to further identify its important modules. The important modules and targets were screened from the PPI network with a degree cutoff of 2, depth = 100, *k* − core = 2, and node score = 0.2, using the Molecular Complex Detection (MCODE) plug-in Cytoscape. Differences were considered statistically significant at *P* ≤ 0.05.

### 2.5. KEGG Pathway and GO Function Enrichment Analyses

We applied Gene Ontology (GO) enrichment and Kyoto Encyclopedia of Genes and Genomes (KEGG) pathway analyses to systematically analyze the biological functions of drug-disease targets [21, 22]. Metascape (https://metascape.org/) [23], which can perform enrichment analyses of targets, integrate multiple authoritative functional databases such as GO, KEGG, and UniProt, and support annotation and enrichment analysis. We submitted the targets in the network to Metascape to perform GO and KEGG pathway enrichment analyses. *P* ≤ 0.01 was considered statistically significant.

### 2.6. Molecular Docking

In order to verify whether the compound and the target have good binding activity, we selected the hub components of the drug-component-target network and the hub targets of the drug-disease target PPI network to perform molecular docking. We obtained the 3D structure of components and targets from ZINC [24] (https://zinc.docking.org) and Protein Data Bank [25] (PDB, http://www.rcsb.org), respectively. ZINC is a free database of commercially available compounds for virtual screening whereas PDB is a leading global resource for experimental data central to scientific discovery, which provides access to 3D structure data for large biological molecules. Molecular docking was performed using iGEMDOCK (http://gemdock.life.nctu.edu.tw/dock/igemdock.php, Version 2.1) [26], a tool that uses *k*-means and hierarchical clustering methods based on docking sites and compound properties.

## 3. Results

### 3.1. Identification of Active Compounds and Target Prediction of LWDH Pills

A total of 475 compounds from six herbs in LWDH pills were obtained from the TCMSP database. After screening and removing duplicate values using the two key ADME parameters of OB ≥ 30% and DL ≥ 0.18, we obtained 210 active compounds, including 19 compounds of FL, 32 compounds of MDP, 43 compounds of SY, 132 compounds of SZY, two compounds of SDH, and seven compounds of ZX. Finally, we obtained 204 targets of LWDH pills by compiling the corresponding targets of the active compounds of the six herbs using the TCMSP database.

### 3.2. Predicting Target of PD

The number of PD-related targets obtained from the four databases Genecards, OMIM, TTD, and DrugBank was 3827, 526, 89, and 202, respectively. We summarized the obtained targets and removed duplicate values and finally obtained 4333 PD-related targets.

### 3.3. Construction and Analysis of Drug-Components-Targets Network

**Figure 1:**
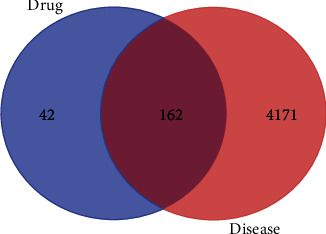
Venn diagram of PD-related targets and targets of LWDH pills.

**Figure 2:**
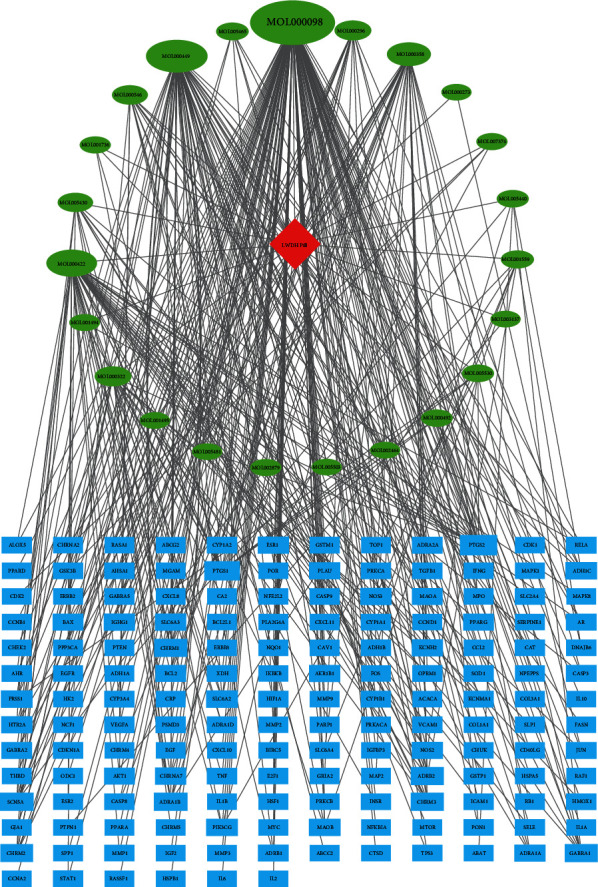
Drug-components-targets network. (The green circular nodes represent components, blue boxes represent drug-disease targets, and diamond node represents LWDH pills; the size of the circular is related to the degree).

On analyzing the identified targets of LWDH pills and PD-related targets using R (to illustrate a Venn diagram; Figure 1), 162 drug-disease targets were obtained. Then, according to the screened drug-disease targets and their pairing relationship with active ingredients, 23 active components of 210 active ingredients that could be targeted to PD-related targets were identified (Table 1). By inputting the relationship between these active components and drug-disease targets into Cytoscape software, we obtained a drug-component-targets network with 186 nodes and 379 edges (Figure 2). We used the network analyzer analysis tool of Cytoscape to analyze the network characteristic parameters to obtain the BC, CC, and degree of each component. The results predicted that quercetin (BC = 0.70528, CC = 0.61056, degree = 117) would be the hub component of LWDH pills in the treatment of PD, followed by stigmasterol (BC = 0.08635, CC = 0.38144, degree = 68), kaempferol (BC = 0.13884, CC = 0.41761, degree = 45), and beta-sitosterol (BC = 0.10312, CC = 0.39278, degree = 31).

### 3.4. PPI Network Construction and Module Screening

**Figure 3:**
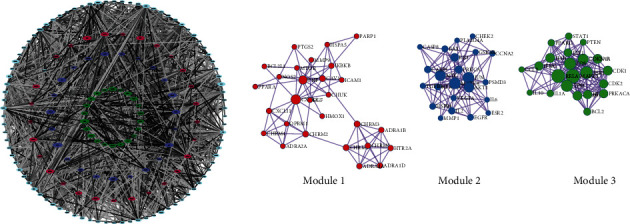
LWDH pills-PD target PPI network and modules.

Based on the results of these drug-disease targets from STRING, we used Cytoscape to construct a PPI network with 160 nodes and 2800 edges (Figure 3). We used the network analysis tool of Cytoscape to analyze the network and adjusted the size of each target in the PPI network according to the degree value. The color of edges is based on the combined score between the targets; the larger the combined score, the darker the color. After obtaining the PPI network, we used the MCODE plug-in to analyze the interaction through the molecular complex detection algorithm and to obtain the modules (Figure 2). According to the *P* value, the biological processes with the three best scores in the modules were retained to describe their functions (Table 2). AKT1, VEGFA, and IL6 in the PPI network had a higher degree value, which suggests that they may be the hub targets of LWDH pills in the treatment of PD.

### 3.5. KEGG Pathway and GO Function Enrichment Analysis

**Figure 4:**
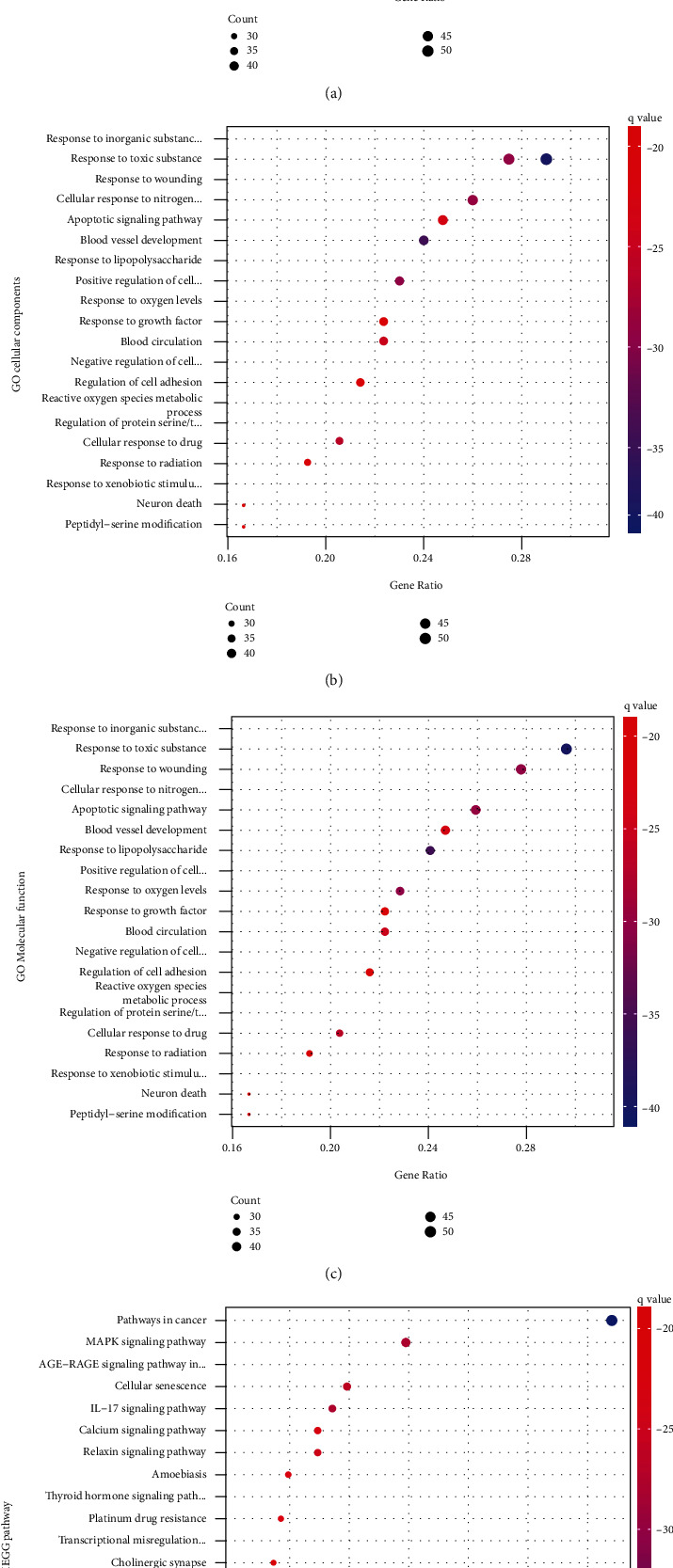
Results of enrichment analysis.

We used the Metascape database to perform enrichment analysis on the above drug-disease targets, including GO biological process (BP), GO cellular component, GO molecular function, and KEGG pathways. Then, we saved the top 20 results for each item and created bubble charts for further analysis (Figure 4). It can be seen that these targets were enriched in many biological processes; the LWDH pills could regulate various biological processes in the body to achieve the purpose of treating PD. Among them, the biological processes most closely related to PD included neuronal death, G protein-coupled amine receptor activity, reactive oxygen species metabolic processes, membrane rafts, MAPK signaling pathways, and cellular senescence.

### 3.6. Molecular Docking

**Figure 5:**
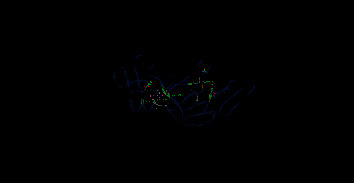
Molecular docking diagram of VEGFA and quercetin.

We screened four hub components as ligands and three hub targets as binding sites for molecular docking. The energy levels of the molecular docking results were all less than -70 (Table 3). Among these components and targets, quercetin had the strongest binding ability with VEGFA (Figure 5).

## 4. Discussion

PD is a common neurodegenerative disease in middle-aged and elderly individuals. Because of its high prevalence, high disability rate, and chronic disease course, it has gradually become an important science and social issue in the field of population and health. DA receptor dysregulation is the pathophysiological basis of PD. However, a clear consensus on its pathogenesis does not exist. Presently, it is believed that factors such as mitochondrial dysfunction caused by oxidative stress, abnormal protein folding caused by endoplasmic reticulum stress, neuroinflammation, the microbiota-gut-brain axis, and the changes of expression of related gene expression are closely related to the occurrence and development of PD [27].

In this study, we retrieved targets of LWDH pills and that of PD from multiple databases and combined them to obtain 162 targets of LWDH pills for the treatment of PD. Then, we constructed a PPI network and screening module and could determine that the genes encoding AKT1, VEGFA, and IL6 are important in this regard. Basic research has shown that AKT1 regulates pathological angiogenesis, vascular maturation, and permeability in vivo [28]. Quesada et al. [29] confirmed through animal experiments that PI3 kinase/Akt activates estrogen and IGF-1 nigral DA, thereby producing a neuroprotective effect in a unilateral rat model of PD. VEGFA is a highly specific vascular endothelial cell growth-promoting factor that promotes the increase of vascular permeability, degeneration of the extracellular matrix, migration, proliferation, and angiogenesis of vascular endothelial cells [30]. Zhang et al. [31] suggested through animal experiments that the miR-339-5p/VEGFA axis plays a role in preventing neuronal apoptosis following intracerebral hemorrhage (ICH) injury. IL6 is a pleiotropic cytokine with a wide range of functions. It regulates the growth and differentiation of a variety of cells, immune response, acute phase response, and hematopoietic function [32]. IL6 is also reported to be a neurotoxic molecule wherein activated microglia can release IL6 and other neurotoxic molecules and affect PD and other neurodegenerative diseases [33, 34]. Furthermore, we screened the modules in the PPI network and described their functions. Module is the area with higher connection density in the network. Module is considered to be biologically significant. It had two meanings. One is a protein complex, in which multiple proteins form a complex and then play a biological role; the other is a functional module, such as proteins located in the same pathway, which interact more closely [35]. Our results showed that the function of the modules is related to G protein-coupled amine receptor activity, MAPK signaling pathway, and apoptosis signaling pathway. Previous studies have shown that DA receptors belong to the family of G protein-coupled receptors (GPCRs), which are regulated by G protein-coupled receptor kinases (GRKs) and arrestins. Research on specific gene knockout mice suggests that GPCRs may be selectively phosphorylated by certain GRK subtypes and selectively bind to certain arrestin subtypes [36, 37]. In the striatum, the D1 receptor mostly binds to arrestin 3, whereas the D2 receptor mostly binds to arrestin 2 [38, 39].

Neuroinflammation is an important cause of DA neuron degeneration. Basic research has shown that p38 mitogen-activated protein kinase (p38MAPK) signaling pathway is also involved in the immune inflammatory response in PD. Additionally, the apoptosis signaling pathway is presently considered one of the pathways of PD pathogenesis. Subsequently, we performed KEGG pathway and GO function enrichment analyses of these targets. According to the *q* value, the most important biological processes were neuron death, G protein-coupled amine receptor activity, reactive oxygen species metabolic process, membrane raft, MAPK signaling pathway, and cellular senescence. Some of them were the same as the biological functions of the modules. Neuronal death is the pathological basis of PD. The metabolic process of reactive oxygen species and membrane rafts may be related to mitochondrial dysfunction and thus participate in the occurrence and development of PD. Astrocytes are the bridge connecting neurons and blood vessels and are involved in activities such as underdevelopment, neurotransmitter transmission, brain metabolism, and blood flow regulation [40, 41]. The maintenance of the mitochondrial respiratory chain function of astrocytes is very important for the energy balance of the brain and the production of antioxidants that protect neurons. Hoekstra et al. [42] found that the expression of DRP1 in astrocytes in the brain of patients with PD was reduced; knockdown of DRP1 in astrocytes cultured in vitro significantly affected mitochondrial morphology and spatial positioning in astrocytes. It may interfere with the uptake of Ca2 + -coupled glutamate to induce hepatotoxicity and affect the survival of PD neurons. When U373 MG human star cells were treated with 1-methyl-4-phenyl-1,2,3,6-tetrahydropyridine (MPTP), the synthesis of mitochondrial respiratory chain complex I was inhibited, causing severe symptoms of PD [43].

Moreover, we constructed and analyzed a drug-component-target network to predict the hub components of LWDH pills in the treatment of PD and their mechanisms. Based on the degree, BC, and CC of the drug-component-target network, we predicted that quercetin, stigmasterol, kaempferol, and beta-sitosterol are the hub components of LWDH pills. Quercetin has antioxidant effects and can prevent oxidative stress-induced cell damage. It can effectively inhibit the transcription activity of the gene promoter encoding cyclooxygenase 2, which catalyzes the conversion of arachidonic acid to prostaglandins (PGs) and other inflammatory molecules and stimulates cell proliferation. It also exerts anti-inflammatory activity effects that are related to its strong antioxidative effect [44]. Quercetin metabolites also exhibit anti-inflammatory effects [45]. Stigmasterol is also an anti-inflammatory and antioxidant molecules. Panda et al. [46] found that stigmasterol can reduce blood glucose concentration by reducing the release of thyroxine and increasing the concentration of insulin in the blood. Additionally, it reduces liver lipid peroxidation and increases the activity of catalase (CAT), superoxide dismutase (SOD), and glutathione (GSH). Pandith et al. [47] found that stigmasterol can significantly reduce the inflammatory enzyme cyclooxygenase-2 (COX-2) expression and inducible nitric oxide (NO) synthesis stimulated by lipopolysaccharide (LPS). The mRNA expression of iNOS, while exerting its anti-inflammatory effect, reduces the release of PGE2 and NO. Basic research shows that kaempferol has antioxidant, antitumor, anti-infection, and other biological activities. Moreover, Wu [48] showed through animal experiments that campers have a certain effect on the structure of mitochondria. It also has an inhibitory effect on the mitochondrial respiratory chain. Beta-sitosterol has anti-inflammatory and antioxidant properties and promotes the proliferation and differentiation of embryonic neural stem cells. Yin et al. [49] reacted beta-sitosterol with organic acids to generate beta-sitosterol-2-naphthoyl ester; they found that it can inhibit the expression of TLR4 and NF-*κ*B that causes SOD in mice with acute liver injury. Additionally, GSH level increased, MDA content decreased, and the expression of NRF-2 and HO-1 was upregulated to inhibit oxidative stress. Liao et al. [50] found that beta-sitosterol can inhibit the production of CAS1 and the activation of the MAPK signaling pathway by inhibiting the activation of the inflammasome NLRP3 in epidermal cells and macrophages, leading to increased production of TNF-*α* and IL-1 beta, and activation of the MAPK signaling pathway in cells. The production of IL6 and IL8 was significantly reduced, thus, exerting an anti-inflammatory effect. Furthermore, beta-sitosterol in the diet can smoothly pass through the blood-brain barrier and can be deposited on the cell membrane. Mahmoudi et al. [51] found that treating embryonic neural stem cells with beta-sitosterol-containing Alyssum saxatile (Hologram) upregulated the expression of NOTCH1, HES-1, KI-67, and NICD, which promoted the proliferation and differentiation of the treated cells.

Finally, we performed molecular docking of the hub components and hub targets using iGEMDOCK to verify whether the compound and target have good binding activity. iGEMDOCK can determine the degree of binding between compounds based on the level of energy. The lower the binding energy, the stronger the binding ability of the action. The energy levels of the molecular docking results were all less than -70. This indicates that the hub active ingredients of LWDH pills have good binding activity with the hub targets of PD.

## 5. Conclusions

In summary, based on NP, this study explained the effective active ingredients of LWDH pills and their related targets and pathways for the treatment of PD. We explained its multicomponent, multitarget, and multibiological pathway treatment for PD. The hub components may be quercetin, stigmasterol, kaempferol, and beta-sitosterol, and probably through pairing core targets such as AKT1, VEGFA, and IL6, to regulate neuronal death, G protein-coupled amine receptor activity, reactive oxygen species metabolic processes, membrane rafts, MAPK signaling pathways, and cellular senescence that can play a role in the treatment of PD. Moreover, we proved that the hub component has a good combination with the hub target through molecular docking. This provides a reference for exploring the pharmacological effects of LWDH pills. Because data on bioinformatics inventories are limited, all the active ingredients and targets for LWDH pills are not available, accounting for the present study's limitations. Our next step is to use clinical samples or animals for validating our findings and understanding further in this regard.

## Figures and Tables

**Table 1:** Characteristic parameters of network nodes for the main active ingredients of LWDH Pills.

Mol ID	Molecule name	Attribution herbs	Betweenness centrality	Closeness centrality	Degree
MOL000098	Quercetin	MDP	0.70528	0.61056	117
MOL000449	Stigmasterol	SY,SZY,SDH	0.08635	0.38144	68
MOL000422	Kaempferol	MDP	0.13884	0.41761	45
MOL000358	Beta-sitosterol	SZY	0.10312	0.39278	31
MOL000296	Hederagenin	FL	0.03611	0.36926	16
MOL000322	Kadsurenone	SY	0.04245	0.36926	16
MOL000546	Diosgenin	SY	0.05697	0.36634	14
MOL005430	Hancinone C	SY	0.03033	0.36489	13
MOL001559	Piperlonguminine	SY	0.01517	0.35783	8
MOL005465	AIDS180907	SY	0.02240	0.35645	7
MOL005440	Isofucosterol	SY	0.02585	0.35509	6
MOL005530	Hydroxygenkwanin	SZY	0.00230	0.35509	6
MOL000492	(+)-catechin	MDP	0.00614	0.35373	5
MOL001736	(-)-taxifolin	SY	0.00070	0.35238	4
MOL002879	Diop	SZY	0.00091	0.35238	4
MOL007374	5-[[5-(4-methoxyphenyl)-2-furyl]methylene]barbituric acid	MDP	0.01481	0.35238	4
MOL001494	Mandenol	SZY	0.00027	0.35104	3
MOL000273	(2R)-2-[(3S,5R,10S,13R,14R,16R,17R)-3,16-dihydroxy-4,4,10,13,14-pentamethyl-2,3,5,6,12,15,16,17-octahydro-1H-cyclopenta[a]phenanthren-17-yl]-6-methylhept-5-enoic acid	FL	0.00273	0.34972	2
MOL001495	Ethyl linolenate	SZY	0.00013	0.34972	2
MOL002464	1-Monolinolein	ZX	0.00013	0.34972	2
MOL003137	Leucanthoside	SZY	0.01081	0.34972	2
MOL005481	2,6,10,14,18-pentamethylicosa-2,6,10,14,18-pentaene	SZY	0.00004	0.34972	2
MOL005503	Cornudentanone	SZY	0.00004	0.34972	2

**Table 2:** LWDH pills-PD targets PPI network function description.

Module	Function description	Log10 (*P*)
Module 1	G protein-coupled amine receptor activity	-20.7
Module 2	MAPK signaling pathway	-13.88
Module 3	Apoptotic signaling pathway	-10.02

**Table 3:** Target component docking results of LWDH pills on PD.

Target name	PDB ID	Compound	Energy level
AKT1	1UNQ	Beta-stigmasterol	-84.1854
		Kaempferol	-86.4579
		Quercetin	-88.1831
		Stigmasterol	-85.0215
VEGFA	1MKK	Beta-stigmasterol	-91.6717
		Kaempferol	-95.6635
		Quercetin	-94.2776
		Stigmasterol	-104.27
IL6	1ALU	Beta-stigmasterol	-73.9486
		Kaempferol	-77.918
		Quercetin	-79.0388
		Stigmasterol	-73.4149

## Data Availability

The data is available from TCMSP (http://tcmspw.com/tcmsp.php), Durg Bank (https://go.drugbank.com), Gene Cards (https://www.genecards.org), TTD (http://db.idrblab.net/ttd/), and OMIM (https://omim.org/).
